# Identified the Synergistic Mechanism of *Drynariae Rhizoma* for Treating Fracture Based on Network Pharmacology

**DOI:** 10.1155/2019/7342635

**Published:** 2019-10-20

**Authors:** Haixiong Lin, Xiaotong Wang, Ligang Wang, Hang Dong, Peizhen Huang, Qunbin Cai, Yingjie Mo, Feng Huang, Ziwei Jiang

**Affiliations:** ^1^The First School of Clinical Medicine, Guangzhou University of Chinese Medicine, Guangzhou 510405, China; ^2^South China Research Center for Acupuncture and Moxibustion, Medical College of Acu-Moxi and Rehabilitation, Guangzhou University of Chinese Medicine, Guangzhou 510006, China; ^3^Department of Orthopaedics, Shenzhen Pingle Orthopedics Hospital & Shenzhen Pingshan District Chinese Medicine Hospital, Guangzhou University of Chinese Medicine, Shenzhen 518000, China; ^4^The First Affiliated Hospital of Guangzhou University of Chinese Medicine, Guangzhou 510405, China; ^5^Dongguan Hospital of Traditional Chinese Medicine, Dongguan 523127, China

## Abstract

**Background:**

*Drynariae Rhizoma* (DR) has been widely used in the prevention and treatment of various fractures. However, the specific mechanisms of DR's active ingredients have not been elucidated. The purpose of this study was to explore the synergistic mechanisms of DR for treating fracture.

**Methods:**

A network pharmacology approach integrating ingredient screening, target exploration, active ingredients-gene target network construction, protein-protein interaction network construction, molecular docking, gene-protein classification, gene ontology (GO) functional analysis, KEGG pathway enrichment analysis, and signaling pathway integration was used.

**Results:**

This approach identified 17 active ingredients of DR, interacting with 144 common gene targets and 143 protein targets of DR and fracture. NCOA1, GSK3B, TTPA, and MAPK1 were identified as important gene targets. Five most important protein targets were also identified, including MAPK1, SRC, HRAS, RXRA, and NCOA1. Molecular docking found that DR has a good binding potential with common protein targets. GO functional analysis indicated that common genes involve multiple processes, parts and functions in biological process, cellular component, and molecular function, including positive regulation of transcription from RNA polymerase II promoter, signal transduction, cytosol, extracellular exosome, cytoplasm, and protein binding. The KEGG pathway enrichment analysis indicated that common gene targets play a role in repairing fractures in multiple signaling pathways, including MAPK, PI3K/AKT, Ras, and VEGF signaling pathways. MAPK and PI3K/AKT signaling pathways were involved in osteoblast formation, Ras signaling pathway was involved in enhancing mesenchymal stromal cell migration, and VEGF signaling pathway was involved in angiogenesis.

**Conclusion:**

The study revealed the correlation between DR and fracture and the potential synergistic mechanism of different targets of DR in the treatment of fractures, which provides a reference for the development of new drugs.

## 1. Introduction

Fracture is a common and frequent disease that occurs in patients with various injuries or osteoporosis [1]. In China, the population-weighted incidence of traumatic fractures of the legs, arms, or trunk in 2014 was 3.21 per 1,000 people (95% CI 2.83–3.59) [2]. Osteoporotic fractures are estimated to account for half of all fractures by 2050, and the estimated cost of osteoporotic hip fractures worldwide may reach $131 billion [3]. Therefore, the study of drugs for the prevention and treatment of fractures plays an important role in promoting patient health and reducing family economic pressure.

Recently, DR, one of the plants from Davalliaceae and Davallia Sm., has been widely used in the prevention and treatment of various fractures due to excellent treatment, low side effects, extensive use, and safety [4]. Animal experiments have confirmed that DR could alter the bone histomorphology and increase the number of trabeculae by 10% [5], and its osteogenesis is related to Runx2 and BMP-2 signaling pathways [6]. In addition, it is believed that the various ingredients contained in an herb could regulate multiple targets in different signaling pathways and produce synergistic therapeutic effects [7]. However, such research has not been carried out in the treatment of fractures with DR.

Network pharmacology based on systems biology and polypharmacology has achieved a paradigm shift from “one drug, one goal” to “multi-ingredient therapy, biological network,” which has attracted the attention of Chinese medicine researchers and has been recognized as an effective tool for elucidating multiple components, targets, synergistic effects, and mechanisms of Chinese medicine [8–10]. It is reported that network pharmacology predicts the clinical efficacy, pathways, and side effects of drugs by constructing drug-drug networks, disease-drug networks, and disease-disease networks, providing valuable information for improving the clinical efficacy, reducing toxicity, and elucidating multimechanisms of drugs [11]. For example, Wang Nani found that Er-Xian Decotion has 13 main components closely related to 65 osteoporosis-related targets by using network pharmacology, thereby constructing Er-Xian Decotion component-osteoporosis target network and potential antiosteoporosis mechanism [12]. Yueying et al. identified 108 compounds, 86 potential targets, and 47 signal transduction pathways that Danshiliuhao Granule regulates liver fibrosis by the network pharmacology method, which reflects the multicomponent, multitarget, and multichannel characteristics of Chinese herbal medicine in antiliver fibrosis [13]. Therefore, in order to reveal the relationship between fracture and the active ingredients involved in the DR, we conducted network pharmacology to achieve this goal from protein and gene level. We collected the information of targets from active ingredients in DR and targets of fracture from several databases, respectively, and used network pharmacology to explore the potential synergistic mechanisms of DR for treating fracture.

## 2. Materials and Methods

### 2.1. Screening of Active Ingredients of *Drynariae Rhizoma*

Traditional Chinese Medicine Systems Pharmacology (TCMSP, http://lsp.nwu.edu.cn/, Version 2.3) Database and Analysis Platform includes chemicals, targets, and drug-target-disease networks, as well as pharmacokinetic properties involving oral bioavailability, druglikeness, blood-brain-barrier, and so on [14]. There were 71 compounds of DR which were obtained from the TCMSP. The potential active ingredients of DR for treating fracture were screened according to their oral bioavailability (OB) ≥30% and druglikeness (DL) ≥0.18 recommended by TCMSP.

### 2.2. Obtaining the Chemical Structure of Active Ingredients

The structure of the potential active ingredients of DR was downloaded from TCMSP and stored in mol2 format. If there was no chemical structure, the PubChem compound was put into the PubChem (https://pubchem.ncbi.nlm.nih.gov/) to download a chemical structure and save it in sdf format, or the PubChem compound was put into the Zinc database (https://zinc.docking.org/) to download a chemical structure and save it in mol2 format. The related SMILES of potential active ingredients was received from TCMSP or PubChem or Zinc database. Then, the SMILES was put into the Swiss Target Prediction database (http://www.swisstargetprediction.ch/) to obtain the related drug target and save it.

### 2.3. Gene Targets of *Drynariae Rhizoma*

The DRAR-CPI server (http://cpi.bio-x.cn/drar, update in 2017-7-26) has a collection of drug molecules and targetable human proteins [15]. When submitting a drug molecule, the server docks the drug uploaded by users with the three-dimensional structure of all protein targets in the database, scores, and ranks them with the affinity scoring function based on the protein-ligand interaction, thereby predicting the potential protein targets of human-targetable drugs [15, 16]. This affinity score is called *Z*-score in the DRAR-CPI server [17]. Protein-ligand interaction with *Z*-score <−0.5 was recommended by DRAR-CPI as a potential protein target for human-targetable drugs [16]. We uploaded the potential active ingredients of DR in mol2 or sdf format and used *Z*-score <−0.5 to select potential protein targets for DR. A total of 1760 proteins with *Z*-score <−0.5 and 355 protein targets were obtained after deletion of the duplicate data. The PDB ID of the protein targets were inputted into UniProt KB (http://www.uniprot.org/uniprot/) of the UniProt database, and the “popular organisms” was selected as human to obtain the gene targets associated with the potential active ingredient of DR.

### 2.4. Gene Target Prediction for *Drynariae Rhizoma* to Treat Fractures

The following electronic databases were searched to identify the genes related to fractures: Genetic Association Database (https://geneticassociationdb.nih.gov/), Therapeutic Targets Database (http://bidd.nus.edu.sg/BIDD-Databases/TTD/TTD.asp), PharmGkb database (https://www.pharmgkb.org/), GeneCards database (http://www.genecards.org/), and OMIM database (http://www.ncbi.nlm.nih.gov/omim). Then, the duplicate data and false-positive genes were deleted. Finally, the Venny tool (http://bioinfogp.cnb.csic.es/tools/venny/index.html, Version 2.1) was used to identify the common gene targets of DR and fracture, which may be the potential targets for DR to treat fractures.

### 2.5. Constructing the Ingredient-Target Network of *Drynariae Rhizoma*

The common gene targets of DR and fracture were introduced into the Cytoscape software (Version 3.4.0) to construct an ingredient-target network of *Drynariae Rhizoma* and analyze the topology properties of the network, including degree, betweenness centrality, and closeness centrality [18]. The degree describes the number of connections to a node in the network, indicating interaction with other nodes in the network. Betweenness centrality measures the proportion of a node between shortest paths among other nodes, suggesting the importance of nodes in maintaining network tightness. Closeness centrality indicates the degree of nodes close to the “center” of the network. A node with high degree, betweenness centrality, and closeness centrality values means that it plays a very important role in the network [18].

### 2.6. Constructing Protein-Protein Interaction (PPI) of *Drynariae Rhizoma*

The String database (https://string-db.org/, Version 10.5) is a database containing known and predicted PPIs, which collect and integrate a large number of protein interactions involving 9,643,763 proteins and 1,380,838,440 interactions, including experimental data and interactive prediction data derived from bioinformatic methods [19]. Common gene targets of DR and fracture were imported into the STRING database, and the species were set to humans for PPIs. Then, the highest confidence was set to 0.9 in the minimum required interaction score and the results were updated. The TSV format of the updated results were downloaded. Then, node1, node2, and combined scores were extracted and imported into the Cytoscape software to create a PPI network, and the network was analyzed as follows: Step 1: analyze the topology properties of the network: cytoscape ⟶ tools ⟶ network analyzer ⟶ network analysis ⟶ analyze network, save the CSV format of the network result and extract the degree value. Step 2: create a network map according to the degree: cytoscape ⟶ tool ⟶ network analyzer ⟶ network analysis ⟶ generate style from statistics ⟶ map node size to degree ⟶ map node color to degree  and save the PPI network map.

### 2.7. Molecular Docking

SystemsDock (http://systemsdock.unit.oist.jp, Version 2.0) is a web server for network pharmacology-based prediction and analysis that could be used to illustrate the role of ligands on a complex molecular network [20]. It evaluates the protein-ligand binding potential of molecular docking by combining docking with the intelligence (dock-IN) score. The dock-IN score is the negative logarithm of the experimental dissociation/inhibition constant (pKd/pKi), which ranges from 0 to 10, indicating weak to strong binding [20]. It is believed that the docK-IN score above 4.25 indicates a slight binding potential between the protein and ligand; a value greater than 5.0 indicates a moderate binding potential, and a value greater than 7.0 indicates a strong binding potential [16]. We extracted the top 5 proteins with the highest degree value in the PPI network. The proteins that were recognized by systemsDock docked with the potential active ingredients of DR to receive the dock-IN score. The results were saved, and their dock-IN score was analyzed to assess the binding potentials between the potential active ingredients of DR and protein targets.

### 2.8. GO Functional Analysis and KEGG Pathway Enrichment Analysis

GO (http://www.geneontology.org) is widely used for annotation of gene function, providing detailed annotations of gene function in terms of biological process (BP), cellular component (CC), and molecular function (MF), respectively [21]. Database for annotation, visualization, and integrated discovery (David, https://david.ncifcrf.gov/, Version 6.8) is a functional genomic annotation database that provides bioinformatics annotation for genes or proteins based on the gene annotation function of the GO database and the signaling pathway information of the KEGG database [22]. We performed GO functional analysis and KEGG pathway enrichment analysis in the David database. The procedure was as follows: Step 1: paste the common gene targets of DR and fracture list. Step 2: select “OFFICIAL_GENE_SYMBOL” in “Select Identifier.” Step 3: select “Gene List” in “List Type.” Step 4: select “*Homo sapiens*” in species. Step 5: submit list. Step 6: download the results of BP, CC, and MF in the gene ontology. Step 7: download the results of KEGG pathway in the pathways. Step 8: targets with *P* < 0.05 were screened and sorted by count (number of targets), and the top-ranked biological processes or KEGG pathways were extracted. Step 9: BP, CC, and MF were designed using GraphPad Prism 5.0 software. The KEGG pathways were designed by the advanced bubble chart of the omicshare tool (http://omicshare.com/tools/Home/Soft/getsoft/type/index).

### 2.9. Collect Protein Class Corresponding to Common Gene Targets

DisGeNET (http://www.disgenet.org/web/DisGeNET/menu, Version 5.0) is a discovery platform that contains one of the largest publicly available genes and variants associated with human disease. It could be used to analyze the properties of disease genes and investigate the molecular basis of specific diseases and their comorbidities, as well as adverse drug reactions [23]. We used the search function of the DisGeNET platform to retrieve the protein class corresponding to common gene targets.

### 2.10. Pathway Integration

We used the KEGG Mapper tool in the KEGG database (http://www.kegg.jp/) to retrieve some pathways of DR for fractures and then integrate into a final pathway map. The procedure was as follows: Step 1: used the UniProt KB search function of the UniProt database to retrieve the UniprotID of the common gene targets. Step 2: import the UniProt ID of the common gene targets. Step 3: set the parameters: search against: hsa, primary ID: NCBI-UniProt ID, and examples: *Homo sapiens* pathway. Step 4: download the PI3K-AKT, MAPK, Ras, and VEGF signaling pathways. Step 5: integrate the signal path.

## 3. Results

### 3.1. Active Ingredients of *Drynariae Rhizoma*

A total of 71 ingredients of DR were retrieved from TCMSP, and 18 active ingredients were screened according to the biological functions of DR. However, marioside_qt (Molecule ID: MOL009087) was removed because it could not be recognized by the PubChem or Zinc database. The remaining 17 active ingredients are shown in Table 1, including (2R)-5,7-dihydroxy-2-(4-hydroxyphenyl)chroman-4-one, aureusidin, eriodictyol (flavanone), stigmasterol, beta-sitosterol, kaempferol, naringenin, (+)-catechin, eriodictyol, digallate, luteolin, 22-stigmasten-3-one, cyclolaudenol acetate, cycloartenone, cyclolaudenol, davallioside A_qt, and xanthogalenol.

### 3.2. Gene Target Prediction

**Figure 1:**
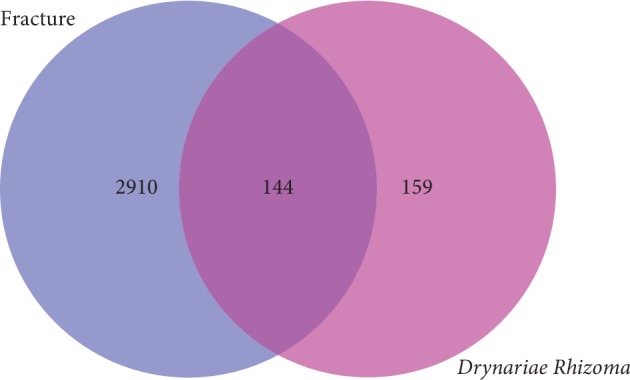
Venn diagram of common gene target screening for fracture and *Drynariae Rhizoma*.

A total of 303 gene targets associated with the potential active ingredients of DR were retrieved in the UniProt database. A total of 3,173 fracture-related genes were received, and 3,054 genes remained after deletion of the duplicate and false-positive genes. Common gene target screening for fracture and DR are shown in Figure 1. A total of 144 common gene targets of DR and fracture were received, indicating the potential targets for DR to treat fractures, as shown in Table 2.

### 3.3. Ingredient-Target Network of *Drynariae Rhizoma*

**Figure 2:**
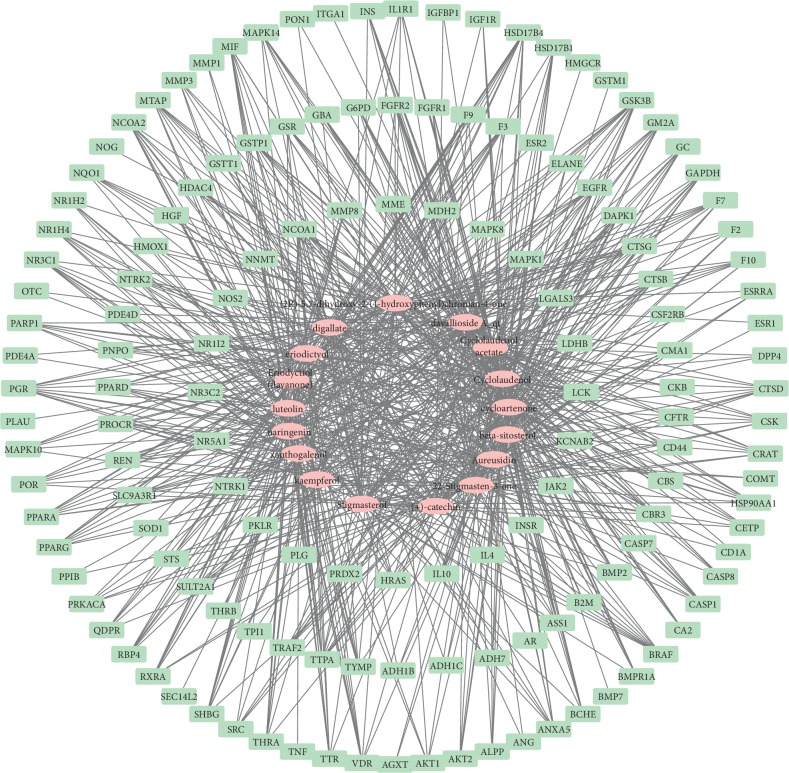
Ingredient-target network of *Drynariae Rhizoma*. *Note*. The pink oval nodes (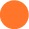) are the main active ingredients of *Drynariae Rhizoma*, and the light green rectangle (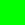) is the potential target for treating fracture of *Drynariae Rhizoma*.

The active ingredients and gene targets of DR was inputted into Cytoscape software to construct the ingredient-target network, as shown in Figure 2. In the network, the pink oval nodes represent the main active ingredients of DR, and the light green rectangle nodes represent the potential gene targets for DR to treat fractures. The line represents the correlation between the active ingredients of DR and the gene targets. There are 161 nodes and 774 lines in the network. An active ingredient could be linked to different gene targets, and a gene target could be linked to different active ingredients, suggesting the multicomponent and multitarget characteristics of DR. The topology properties of active ingredients of DR are shown in Supplementary Table 1. Both cyclolaudenol and cycloartenone were linked to a maximum number of gene targets, with 70 (48.61%) different gene targets. Cyclolaudenol acetate was linked to 54 (37.5%) different gene targets. Xanthogalenol was linked to a minimum number of gene targets for a total of 29 (20.14%). In addition, there were four gene targets with the top four degree values, betweenness centrality, and closeness centrality at the same time (Table 3), which were NCOA1, GSK3B, TTPA, and MAPK1.

### 3.4. Protein-Protein Interaction of *Drynariae Rhizoma*

**Figure 3:**
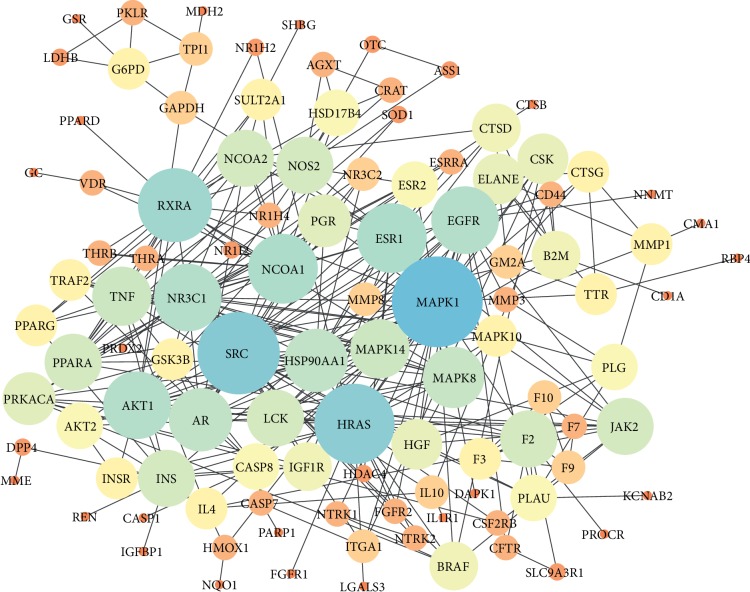
Protein-protein interaction network of *Drynariae Rhizoma*. *Note*. The size and the color of the node represents the value of the degree (yellow ⟶ orange ⟶ blue indicates that the degree value is from low to high, and the small circle represents a low degree value).

The PPI network of DR is shown in Figure 3. In the network, the node represents the protein, and the size and color of the node represent the value of the degree. The larger the node and the brighter the color (yellow to blue), the greater the value of the degree. The line indicates the association between proteins. Results showed that there were 143 nodes and 315 lines.

Degree in the network indicates the number of proteins that a protein has interacting with. In other words, top-degree protein targets screened in PPI plays a pivotal role in the treatment of fractures with DR. Five important protein targets with top degree of DR were identified in the PPI network and are shown in Table 4. They were MAPK1, SRC, HRAS, RXRA, and NCOA1.

### 3.5. Molecular Docking

Three important protein targets with top degree of DR were identified by SystemsDock, including SRC, RXRA, and NCOA1. Dock-IN score of these three proteins docked with 17 active ingredients of DR are shown in Table 5. Molecular docking results showed that there were 17 (33.33%) with a dock-IN score greater than 7.0, 24 (47.06%) with a dock-IN score between 7.0 and 5.0, 8 (15.69%) with a dock-IN score between 5.0 and 4.25, and 2 (3.92%) with a dock-IN score less than 4.25.

### 3.6. Gene Ontology (GO) Functional Analysis and KEGG Pathway Enrichment Analysis

**Figure 4:**
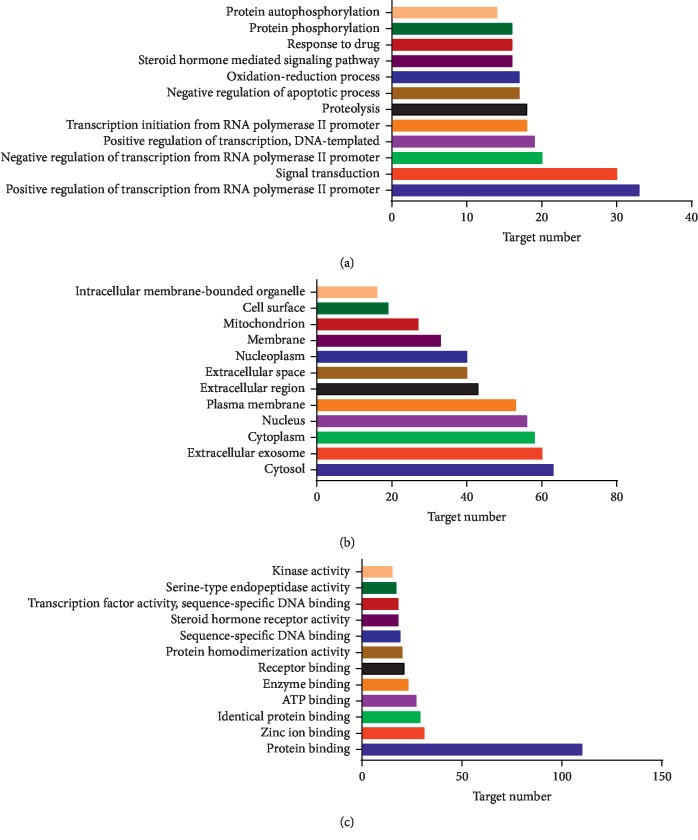
Enriched gene ontology terms of potential therapeutic fracture targets from main active ingredients of *Drynariae Rhizoma*. *Note*. (a) Biological process (BP), (b) cellular component (CC), and (c) molecular function (MF).

Enriched gene ontology terms for BP, CC, and MF of potential therapeutic fracture targets from the main active ingredients of DR are shown in Figure 4. In the BP (Figure 4(a)), positive regulation of transcription from RNA polymerase II promoter involved 33 (22.92%) potential therapeutic fracture targets, signal transduction involved 30 (20.84%) potential therapeutic fracture targets, negative regulation of transcription from RNA polymerase II promoter involved 20 (13.89%) potential therapeutic fracture targets, positive regulation of transcription and DNA-template involved 19 (13.19%) potential therapeutic fracture targets, and transcription initiation from RNA polymerase II promoter involved 18 (12.5%) potential therapeutic fracture targets. In the CC (Figure 4(b)), cytosol involved 63 (43.75%) potential therapeutic fracture targets, extracellular exosome involved 60 (41.67%) potential therapeutic fracture targets, cytoplasm involved 58 (40.28%) potential therapeutic fracture targets, nucleus involved 56 (38.89%) potential therapeutic fracture targets, and plasma membrane involved 53 (36.81%) potential therapeutic fracture targets. In the MF (Figure 4(c)), protein binding involved 110 (76.39%) potential therapeutic fracture targets, zinc ion binding involved 31 (21.53%) potential therapeutic fracture targets, identical protein binding involved 29 (20.14%) potential therapeutic fracture targets, ATP binding involved 27 (18.75%) potential therapeutic fracture targets, and enzyme binding involved 23 (15.97%) potential therapeutic fracture targets.

**Figure 5:**
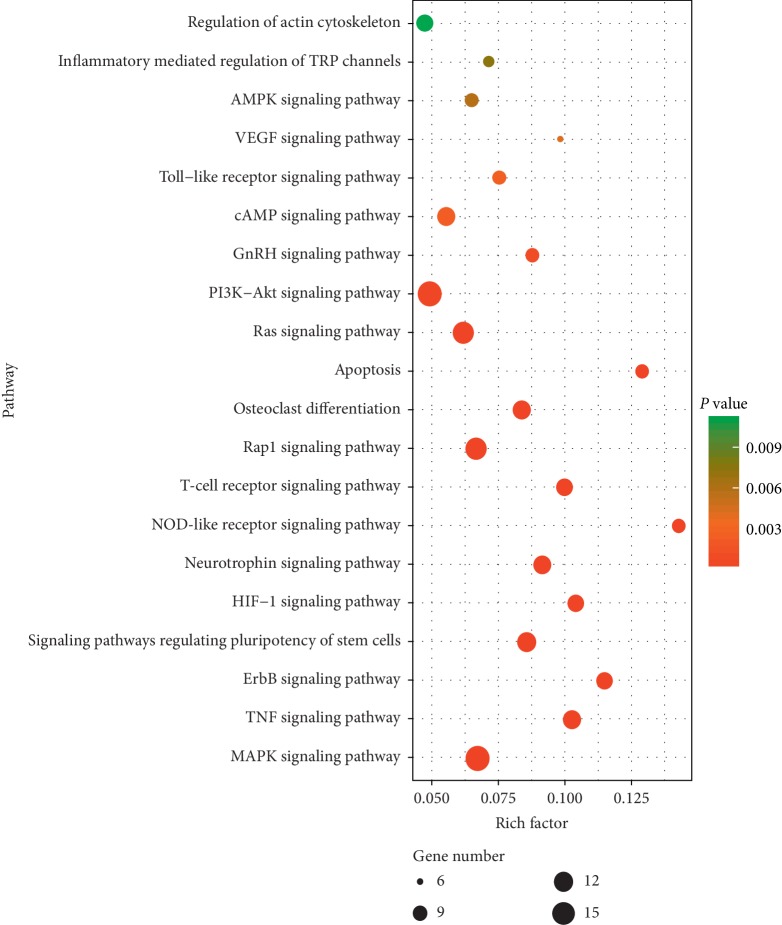
Enriched KEGG pathways of potential targets for treating fracture from main active ingredients of *Drynariae Rhizoma*.

Enriched KEGG pathways of potential targets for treating fracture from the main active ingredients of DR are shown in Figure 5. The MAPK signaling pathway was identified as an important signaling pathway involving 17 (11.81%) potential therapeutic fracture targets with *P*=7.82 × 10^−6^. The PI3K-Akt signaling pathway involved 17 (11.81%) potential therapeutic fracture targets, the Rap1 signaling pathway involved 14 (9.72%) potential therapeutic fracture targets, the Ras signaling pathway involved 14 (9.72%) potential therapeutic fracture targets, and the signaling pathways regulating pluripotency of stem cells involved 12 (9.03%) potential therapeutic fracture targets.

### 3.7. Protein Class Corresponding to Common Gene Targets

The protein class corresponding to potential targets for treating fracture from the main active ingredients of DR is presented in Table 6. The results showed that DR treatment of the fracture process involved a variety of substances, such as signaling molecule, transcription factor, receptor, enzyme modulator, chaperone, cell adhesion molecule, protein (transporter, transfer protein, carrier protein, calcium-binding protein, defense protein, and immune protein), enzyme modulator, and enzymes (oxidoreductase, kinase, phosphatase, hydrolase, ligase, protease, isomerase, lyase, enzyme regulator, and transferase).

### 3.8. Signaling Pathway Integration

**Figure 6:**
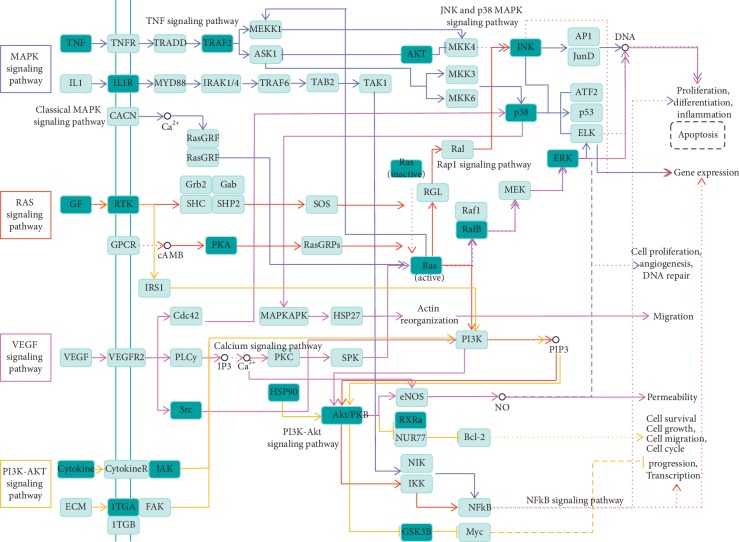
Antifracture pathways of potential targets for treating fracture from main active ingredients of *Drynariae Rhizoma*. *Note*. The arrow (⟶) indicates the promoting effect, the T-arrows (⊣) indicate the inhibition, and the arrows of different colors represent different signaling pathways. The targets of the signaling pathway were marked as light blue, and the potential targets of main active ingredients of DR for treating fracture were marked as dark blue.

Four pathways associated with the potential targets of DR main active ingredients for treating fracture are presented in Figure 6. The arrow (⟶) indicates the promoting effect, the T-arrows (⊣) indicate the inhibition, and the arrows of different colors represent different signaling pathways. The targets of the signaling pathway were marked as light blue, and the potential targets of DR main active ingredients for treating fracture were marked as dark blue. There were 21 (14.58%) potential targets of main active ingredients of DR for treating fracture in the PI3K-AKT, MAPK, Ras, and VEGF signaling pathways, indicating that the fracture targets play a role in these signaling pathways. In addition, some targets play a role in a variety of signaling pathways, such as Ras, RafB, AKT/PKB, PI3K, ERK, and JNK.

## 4. Discussion

In order to reveal the relationship between fracture and the active ingredients involved in the DR, we predicted the mechanism of DR treatment fractures by constructing a biological network of interactions between active ingredients and common gene targets and common protein targets from a molecular level. A total of 17 active ingredients of DR were received in our study, including (2R)-5,7-dihydroxy-2-(4-hydroxyphenyl)chroman-4-one, aureusidin, eriodictyol (flavanone), stigmasterol, beta-sitosterol, kaempferol, naringenin, (+)-catechin, eriodictyol, digallate, luteolin, 22-stigmasten-3-one, cyclolaudenol acetate, cycloartenone, cyclolaudenol, davallioside A_qt, and xanthogalenol. Most of them were polyphenolic compounds, which are also called flavonoids. Flavonoids are considered to be the main active ingredients of DR and have been reported to reduce bone loss in ovariectomized rats [24]. In addition, Kang Suk-Nam finds that the total phenolics and flavonoids of DR are better extracted with 70% ethanol instead of water, and this ethanol extraction method also makes these extracts have higher antioxidant activity and in vitro antiosteoporosis effect [25]. In the ingredient-target network, all active ingredients were also identified to bind well to the fracture gene targets, binding to at least 29 (20.14%) different gene targets. Therefore, the 17 active ingredients of DR may have the effect of reducing bone loss and promoting fracture healing.

In our study, 144 common gene targets of DR and fracture were received, and 774 interactions between the active ingredients of DR and common gene targets were found. Some gene targets have been confirmed by clinical trials or animal experiments. For example, Guimarães et al. found that polymorphisms in the FGFR1 and BMP4 genes were associated with fracture nonunion in patients [26]. And our team's previous study also found that the total flavonoids of DP could promote osteogenesis and mineralization in rats with tibial defects by increasing the gene expression of BMP2, BMP4, BMPR1A, and Smadl [27]. In the ingredient-target network, NCOA1, GSK3B, TTPA, and MAPK1 were identified as important gene targets based on degree values, betweenness centrality, and closeness centrality. Qin et al. found that NCOA1 promotes angiogenesis by upregulating HIF1*α*- and AP-1-mediated VEGFa transcription [28]. Galli et al. demonstrated by cell experiments that inhibition of GSK3B could increase cytoplasmic availability of b-catenin, thereby enhancing Wnt classical signaling and osteoblastic differentiation [29]. Fujita et al. found that mice deficient in TTPA developed a high bone mass phenotype in vertebrae and long bones due to lower bone resorption [30]. Matsushita et al. confirmed that MAPK1 (also called ERK2) plays an important role in osteoblast differentiation and osteoclastogenesis [31]. These gene targets are involved in vascularization, osteoblast differentiation, and osteoclastogenesis in fracture repair. Besides, we found that one active ingredient can interact with different gene targets, and one gene target can interact with different active ingredients, which is consistent with the modern drug theory of “multi-ingredient, multitarget” [9].

To identify the interactions of proteins corresponding to common genes, we conducted a PPI network. A total of 143 common protein targets for DR and fracture were received, with 315 PPIs. In addition, MAPK1, SRC, HRAS, RXRA, and NCOA1 were identified as the five most important target proteins. Previous studies have found that MAPK1 and SRC could promote proliferation and differentiation of myeloid cells and inhibit apoptosis [32, 33]. Clinical cases have found that elevated levels of fibroblast growth factor 23 in patients with dysplasia are associated with HRAS mutations [34]. RXRA is an essential cofactor in the action of 1,25-dihydroxyvitamin D, and umbilical cord RXRA methylation was inversely related to offspring bone mineral content [35]. Coronnello et al. found that NCOA1 modulate the estrogen effects in bone, and miR-488-5p overexpression reduces NCOA1 protein levels, thereby reducing bone mineral density [36]. These protein targets are associated with bone growth and angiogenesis in fracture repair. At the same time, we docked SRC, RXRA, and NCOA1 with 17 potential active ingredients of DR and found that 41 (80.39%) had moderate binding potential, suggesting that DR could bind well to fracture-related protein targets.

In order to identify the function of the common gene, we performed GO functional analysis on these genes. The results showed that the common gene involves multiple processes, parts and functions in BP, CC, and MF, which was consistent with existing studies about DR and fracture repair. For example, in the BP, 33 (22.92%) gene targets were involved in positive regulation of transcription from the RNA polymerase II promoter, and 30 (20.84%) gene targets were involved in signal transduction. Previous studies have shown that the promoter activates the polymerase to bind precisely to the template DNA and has the specificity of transcription initiation [37]. The RNA polymerase II promoter responsible for mRNA transcription is the largest and most important class of promoters [37]. This provides conditions for DR to initiate osteogenic targets. Besides, some signal transduction genes have been found in experiments. Song Nan found that VEGFR-2 may play a signal transduction role for naringin, one ingredient of DR, to stimulate angiogenesis and promote fracture healing [38]. In the CC, 63 (43.75%) gene targets were involved in cytosol, 60 (41.67%) gene targets were involved in extracellular exosome, and 58 (40.28%) gene targets were involved in cytoplasm. This indicates that the recovery of the fracture requires the support of various components in the cell, which is consistent with previous studies [39]. In the MF, 110 (76.39%) gene targets were involved in protein binding, suggesting that mutual recognition between proteins has good gene regulation conditions. This is consistent with the protein class corresponding to the potential target. These results were further validated in the protein class corresponding to the common gene. In the protein class, all of these common genes have been found to regulate a variety of fracture-related molecules, such as transcription factors, receptors, enzyme regulators, molecular chaperones, cell adhesion molecules, enzyme, and so on.

In order to identify the synergistic mechanism of DR for fracture, we performed KEGG pathway enrichment analysis and summarized some important signaling pathways, which provides direction for future research. In the KEGG pathway enrichment analysis, 17 (11.81%) gene targets were involved in MAPK signaling pathway, 17 (11.81%) gene targets were involved in PI3K-Akt signaling pathway, 14 (9.72%) gene targets were involved in Ras signaling pathway, and 6 (4.17%) gene targets were involved in VEGF signaling pathway, which suggest that common gene targets play a role in repairing fractures in multiple signaling pathways. MAPK and PI3K/AKT signaling pathways have been demonstrated to promote osteoblastic bone formation [40]. Zhang et al. confirmed that total flavonoids from DR promote the osteogentic differentiation of ciliary neurotrophic factor-modified myoblasts by activating p38 MAPK signaling pathway [41]. Moreover, total flavonoids of DR could promote osteogenic differentiation of rat dental pulp stem cells via the PI3K/Akt pathway [42]. Lin et al. found that the effect of naringin on the healing of fracture may be related to the promotion of the synthesis and secretion of cellular chemokines (CXCL5, CXCL6) and enhancement of mesenchymal stromal cell migration through Ras signaling pathway [43]. In addition, naringin stimulates angiogenesis by regulating the VEGF/VEGFR-2 signaling pathway in rats, thereby promoting fracture healing [38]. However, the mechanism of some active ingredients of DR in the treatment of fractures has not yet been verified. Therefore, we integrated MAPK, PI3K/AKT, Ras, and VEGF signaling pathways to provide a reference for researchers to verify the mechanism of other DR active ingredients in the treatment of fractures.

## 5. Conclusion

We collected the gene and protein targets of fractures and active ingredients of DR and then used network pharmacology to reveal the correlation between drugs and diseases and the potential synergistic mechanism of different targets of DR in the treatment of fractures, which provides a reference for the development of new drugs.

## Figures and Tables

**Table 1:** Main active ingredients in *Drynariae Rhizoma*.

No.	Molecule ID	Molecule name	Chemical formula	Structure	OB (%)	DL
1	MOL001040	(2R)-5,7-Dihydroxy-2-(4-hydroxyphenyl)chroman-4-one	C_15_H_12_O_5_	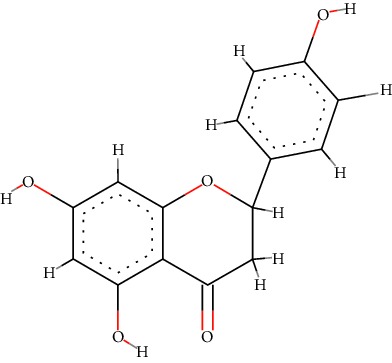	42.36	0.21
2	MOL001978	Aureusidin	C_15_H_10_O_6_	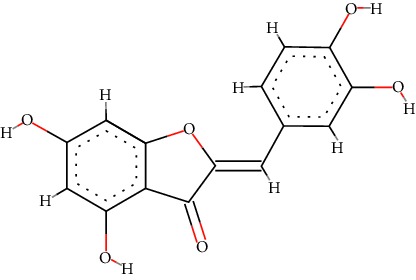	53.42	0.24
3	MOL002914	Eriodictyol (flavanone)	C_15_H_12_O_6_	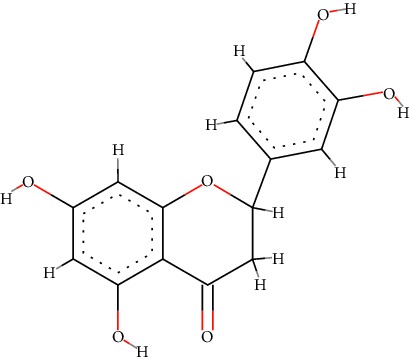	41.35	0.24
4	MOL000449	Stigmasterol	C_29_H_48_O	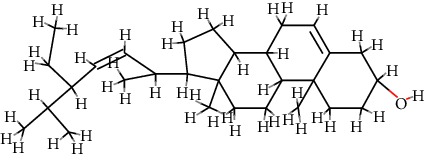	43.83	0.76
5	MOL000358	*β*-Sitosterol	C_29_H_50_O	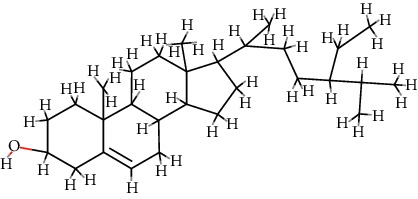	36.91	0.75
6	MOL000422	Kaempferol	C_15_H_10_O_6_	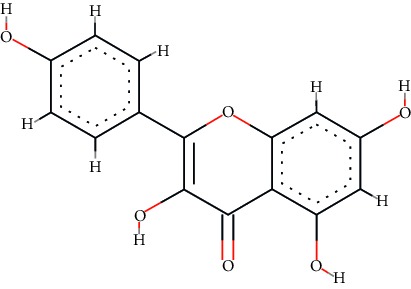	41.88	0.24
7	MOL004328	Naringenin	C_15_H_12_O_5_	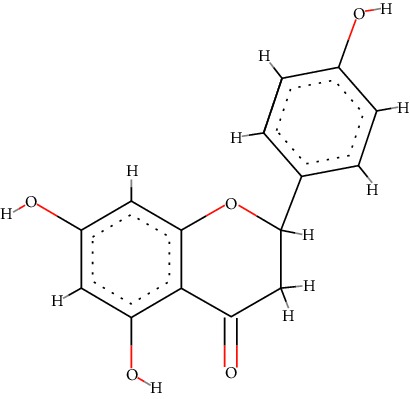	59.29	0.21
8	MOL000492	(+)-Catechin	C_15_H_14_O_6_	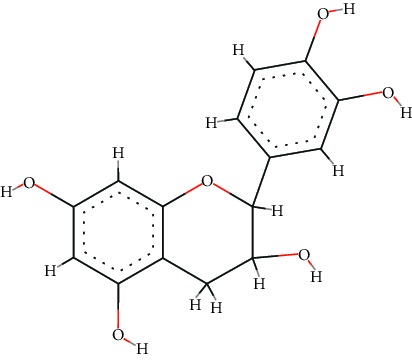	54.83	0.24
9	MOL005190	Eriodictyol	C_15_H_12_O_6_	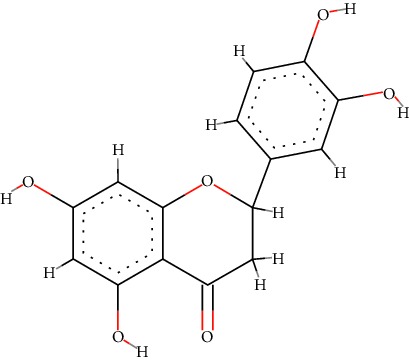	71.79	0.24
10	MOL000569	Digallate	C_14_H_10_O_9_	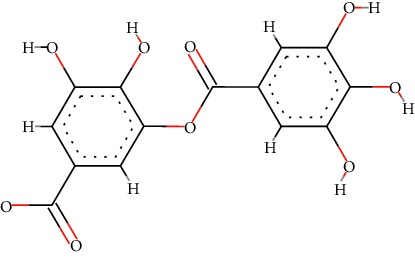	61.85	0.26
11	MOL000006	Luteolin	C_15_H_10_O_6_	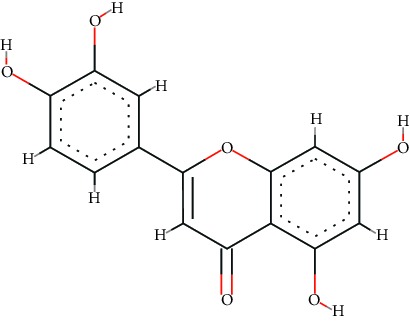	36.16	0.25
12	MOL009061	22-Stigmasten-3-one	C_29_H_48_O	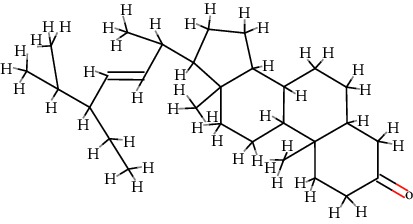	39.25	0.76
13	MOL009063	Cyclolaudenol acetate	C_33_H_54_O_2_	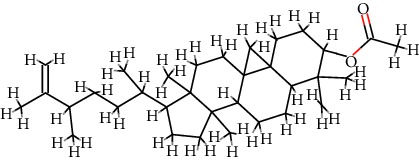	41.66	0.79
14	MOL009075	Cycloartenone	C_30_H_48_O	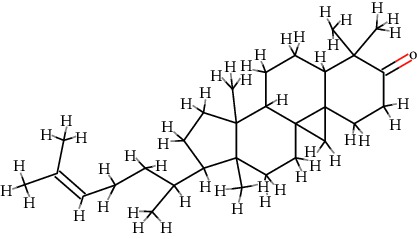	40.57	0.79
15	MOL009076	Cyclolaudenol	C_31_H_52_O	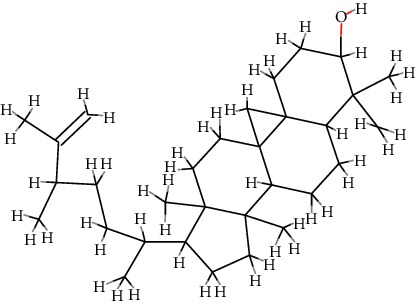	39.05	0.79
16	MOL009078	Davallioside A_qt	C_25_H_29_NO_12_	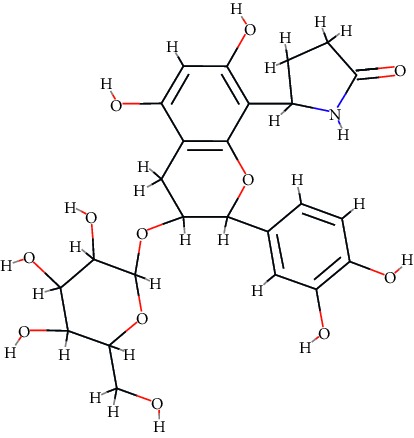	62.65	0.51
17	MOL009091	Xanthogalenol	C_21_H_22_O_5_	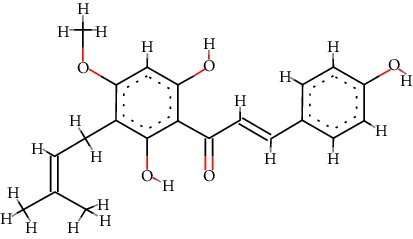	41.08	0.32

**Table 2:** Information of potential gene targets for treating fracture from *Drynariae Rhizoma*.

No.	PDB ID	Gene target
1	1HSZ	ADH1B
2	1HT0	ADH1C
3	1D1T	ADH7
4	1H0C	AGXT
5	3CQW	AKT1
6	1O6L	AKT2
7	2GLQ	ALPP
8	1ANG	ANG
9	1HAK	ANXA5
10	1E3G	AR
11	2NZ2	ASS1
12	1ONQ	B2M
13	1XLV	BCHE
14	1ES7	BMP2
15	1M4U	BMP7
16	1ES7	BMPR1A
17	1UWJ	BRAF
18	1A42	CA2
19	1ICE	CASP1
20	1K86	CASP7
21	2C2Z	CASP8
22	2HRB	CBR3
23	1JBQ	CBS
24	1ONQ	CD1A
25	1POZ	CD44
26	2OBD	CETP
27	1XMI	CFTR
28	3DRB	CKB
29	1NN6	CMA1
30	3BWY	COMT
31	1NM8	CRAT
32	1C8P	CSF2RB
33	1BYG	CSK
34	1CSB	CTSB
35	1LYW	CTSD
36	1CGH	CTSG
37	1JKL	DAPK1
38	2HHA	DPP4
39	1M17	EGFR
40	1H1B	ELANE
41	1R5K	ESR1
42	1QKM	ESR2
43	2PJL	ESRRA
44	1F0R	F10
45	1A3B	F2
46	1Z6J	F3
47	1Z6J	F7
48	1RFN	F9
49	2FGI	FGFR1
50	2PVY	FGFR2
51	2BH9	G6PD
52	1ZNQ	GAPDH
53	1OGS	GBA
54	1J78	GC
55	1PUB	GM2A
56	1J1B	GSK3B
57	1GRE	GSR
58	1XWK	GSTM1
59	11GS	GSTP1
60	2C3Q	GSTT1
61	2VQM	HDAC4
62	1GMN	HGF
63	1HWL	HMGCR
64	1S8C	HMOX1
65	5P21	HRAS
66	1DHT	HSD17B1
67	1ZBQ	HSD17B4
68	1YET	HSP90AA1
69	2OJ9	IGF1R
70	1ZT3	IGFBP1
71	2ILK	IL10
72	1G0Y	IL1R1
73	2CYK	IL4
74	1TYL	INS
75	2AUH	INSR
76	1QCY	ITGA1
77	2B7A	JAK2
78	1ZSX	KCNAB2
79	1QPC	LCK
80	1I0Z	LDHB
81	1KJL	LGALS3
82	1TVO	MAPK1
83	1JNK	MAPK10
84	1A9U	MAPK14
85	1UKI	MAPK8
86	2DFD	MDH2
87	1GCZ	MIF
88	1DMT	MME
89	1HFC	MMP1
90	1QIA	MMP3
91	1JAP	MMP8
92	1SD2	MTAP
93	2P54	NCOA1
94	1MVC	NCOA2
95	2IIP	NNMT
96	1M4U	NOG
97	1NSI	NOS2
98	1KBQ	NQO1
99	1UPV	NR1H2
100	3FXV	NR1H4
101	1NRL	NR1I2
102	1P93	NR3C1
103	2A3I	NR3C2
104	1YOW	NR5A1
105	1WWA	NTRK1
106	1WWB	NTRK2
107	1OTH	OTC
108	1WOK	PARP1
109	2QYK	PDE4A
110	1PTW	PDE4D
111	1ZUC	PGR
112	2VGB	PKLR
113	1VJA	PLAU
114	2PK4	PLG
115	1NRG	PNPO
116	1V04	PON1
117	1B1C	POR
118	2P54	PPARA
119	2J14	PPARD
120	1ZEO	PPARG
121	1CYN	PPIB
122	1QMV	PRDX2
123	2GU8	PRKACA
124	1LQV	PROCR
125	1HDR	QDPR
126	1QAB	RBP4
127	2G1N	REN
128	1MVC	RXRA
129	1OLM	SEC14L2
130	1F5F	SHBG
131	1I92	SLC9A3R1
132	2C9V	SOD1
133	1YOL	SRC
134	1P49	STS
135	1J99	SULT2A1
136	1NAV	THRA
137	1NAX	THRB
138	1A8M	TNF
139	1HTI	TPI1
140	1D0A	TRAF2
141	1OIZ	TTPA
142	1QAB	TTR
143	1UOU	TYMP
144	3CS4	VDR

**Table 3:** Gene targets with the top 4 degree values, betweenness centrality, and closeness centrality.

No.	Gene targets	Degree (rank)	Betweenness centrality (rank)	Closeness centrality (rank)
1	NCOA1	15 (1)	0.038 (1)	0.505 (1)
2	MAPK1	13 (2)	0.023 (3)	0.464 (4)
3	GSK3B	12 (3)	0.028 (2)	0.502 (2)
4	TTPA	12 (4)	0.022 (4)	0.466 (3)

**Table 4:** Five important protein targets with top degree of *Drynariae Rhizoma*.

No.	Degree	PDB ID	Protein target name
1	27	1TVO	MAPK1
2	23	1YOL	SRC
3	22	5P21	HRAS
4	20	1MVC	RXRA
5	18	2P54	NCOA1

**Table 5:** Molecular docking of three important protein targets from *Drynariae Rhizoma*.

Protein target	PDB ID	Ingredients	Dock-IN score
NCOA1	1NQ7	(+)-Catechin	7.111
RXRA	1DSZ	(+)-Catechin	4.624
SRC	1O4R	(+)-Catechin	5.908
NCOA1	1NQ7	(2R)-5,7-Dihydroxy-2-(4-hydroxyphenyl)chroman-4-one	6.694
RXRA	1DSZ	(2R)-5,7-Dihydroxy-2-(4-hydroxyphenyl)chroman-4-one	4.605
SRC	1O4R	(2R)-5,7-Dihydroxy-2-(4-hydroxyphenyl)chroman-4-one	5.783
NCOA1	1NQ7	22-Stigmasten-3-one	8.422
RXRA	1DSZ	22-Stigmasten-3-one	5.553
SRC	1O4R	22-Stigmasten-3-one	5.425
NCOA1	1NQ7	Aureusidin	7.153
RXRA	1DSZ	Aureusidin	4.622
SRC	1O4R	Aureusidin	5.861
NCOA1	1NQ7	Beta-sitosterol	8.34
RXRA	1DSZ	Beta-sitosterol	5.587
SRC	1O4R	Beta-sitosterol	5.374
NCOA1	1NQ7	Cycloartenone	8.427
RXRA	1DSZ	Cycloartenone	5.658
SRC	1O4R	Cycloartenone	5.693
NCOA1	1NQ7	Cyclolaudenol	8.376
RXRA	1DSZ	Cyclolaudenol	5.534
SRC	1O4R	Cyclolaudenol	5.376
NCOA1	1NQ7	Cyclolaudenol acetate	8.422
RXRA	1DSZ	Cyclolaudenol acetate	7.052
SRC	1O4R	Cyclolaudenol acetate	6.364
NCOA1	1NQ7	Davallioside A_qt	7.904
RXRA	1DSZ	Davallioside A_qt	5.779
SRC	1O4R	Davallioside A_qt	5.58
NCOA1	1NQ7	Digallate	4.313
RXRA	1DSZ	Digallate	3.789
SRC	1O4R	Digallate	3.671
NCOA1	1NQ7	Eriodictyol	7.109
RXRA	1DSZ	Eriodictyol	4.628
SRC	1O4R	Eriodictyol	5.821
NCOA1	1NQ7	Eriodictyol (flavanone)	7.113
RXRA	1DSZ	Eriodictyol (flavanone)	4.633
SRC	1O4R	Eriodictyol (flavanone)	5.824
NCOA1	1NQ7	Kaempferol	7.125
RXRA	1DSZ	Kaempferol	4.613
SRC	1O4R	Kaempferol	5.929
NCOA1	1NQ7	Luteolin	7.089
RXRA	1DSZ	Luteolin	4.621
SRC	1O4R	Luteolin	5.847
NCOA1	1NQ7	Naringenin	7.12
RXRA	1DSZ	Naringenin	6.016
SRC	1O4R	Naringenin	5.883
NCOA1	1NQ7	Stigmasterol	8.376
RXRA	1DSZ	Stigmasterol	5.918
SRC	1O4R	Stigmasterol	5.3
NCOA1	1NQ7	Xanthogalenol	5.387
RXRA	1DSZ	Xanthogalenol	7.093
SRC	1O4R	Xanthogalenol	7.142

**Table 6:** The protein class corresponding to potential targets for treating fracture from main active ingredients of *Drynariae Rhizoma*.

No.	Gene name	Protein class
1	ADH1B	Oxidoreductase
2	ADH1C	Oxidoreductase
3	ADH7	Oxidoreductase
4	AGXT	Transferase
5	AKT1	Calcium-binding protein; kinase; transfer/carrier protein; transferase
6	AKT2	Calcium-binding protein; kinase; transfer/carrier protein; transferase
7	ALPP	Hydrolase; phosphatase
8	ANG	None
9	ANXA5	None
10	AR	Nucleic acid binding; receptor; transcription factor
11	ASS1	Ligase
12	B2M	Defense/immunity protein
13	BCHE	None
14	BMP2	Signaling molecule
15	BMP7	Signaling molecule
16	BMPR1A	Kinase; receptor; transferase
17	BRAF	None
18	CA2	None
19	CASP1	Enzyme modulator; hydrolase; protease
20	CASP7	Enzyme modulator; hydrolase; protease
21	CASP8	Enzyme modulator; hydrolase; protease
22	CBR3	None
23	CBS	Hydrolase; isomerase; lyase
24	CD1A	None
25	CD44	None
26	CETP	None
27	CFTR	Transporter
28	CKB	Kinase; transferase
29	CMA1	Hydrolase; protease
30	COMT	Transferase
31	CRAT	Transferase
32	CSF2RB	Receptor
33	CSK	None
34	CTSB	Enzyme modulator; hydrolase; protease
35	CTSD	Hydrolase; protease
36	CTSG	Hydrolase; protease
37	DAPK1	Kinase; transferase
38	DPP4	Enzyme modulator; hydrolase; protease
39	EGFR	None
40	ELANE	Hydrolase; protease
41	ESR1	Nucleic acid binding; receptor; transcription factor
42	ESR2	Nucleic acid binding; receptor; transcription factor
43	ESRRA	Nucleic acid binding; receptor; transcription factor
44	F10	Hydrolase; protease
45	F2	Hydrolase; protease
46	F3	Defense/immunity protein; receptor
47	F7	Hydrolase; protease
48	F9	Hydrolase; protease
49	FGFR1	None
50	FGFR2	None
51	G6PD	Oxidoreductase
52	GAPDH	Oxidoreductase
53	GBA	None
54	GC	Transfer/carrier protein
55	GM2A	Transfer/carrier protein
56	GSK3B	Kinase; transferase
57	GSR	Oxidoreductase
58	GSTM1	None
59	GSTP1	None
60	GSTT1	None
61	HDAC4	None
62	HGF	Hydrolase; protease
63	HMGCR	None
64	HMOX1	Oxidoreductase
65	HRAS	Enzyme modulator
66	HSD17B1	Oxidoreductase
67	HSD17B4	None
68	HSP90AA1	Chaperone
69	IGF1R	None
70	IGFBP1	Enzyme modulator
71	IL10	None
72	IL1R1	Receptor
73	IL4	None
74	INS	None
75	INSR	None
76	ITGA1	None
77	JAK2	None
78	KCNAB2	Oxidoreductase; transporter
79	LCK	None
80	LDHB	Oxidoreductase
81	LGALS3	Cell adhesion molecule; signaling molecule
82	MAPK1	Kinase; transferase
83	MAPK10	Kinase; transferase
84	MAPK14	Kinase; transferase
85	MAPK8	Kinase; transferase
86	MDH2	Oxidoreductase
87	MIF	None
88	MME	Hydrolase; protease
89	MMP1	Hydrolase; protease
90	MMP3	Hydrolase; protease
91	MMP8	Hydrolase; protease
92	MTAP	Transferase
93	NCOA1	Transcription factor; transferase
94	NCOA2	Transcription factor; transferase
95	NNMT	Transferase
96	NOG	None
97	NOS2	None
98	NQO1	None
99	NR1H2	Nucleic acid binding; receptor; transcription factor
100	NR1H4	Nucleic acid binding; receptor; transcription factor
101	NR1I2	Nucleic acid binding; receptor; transcription factor
102	NR3C1	Nucleic acid binding; receptor; transcription factor
103	NR3C2	Nucleic acid binding; receptor; transcription factor
104	NR5A1	Transcription factor
105	NTRK1	None
106	NTRK2	None
107	OTC	None
108	PARP1	None
109	PDE4A	None
110	PDE4D	None
111	PGR	Nucleic acid binding; receptor; transcription factor
112	PKLR	None
113	PLAU	Hydrolase; protease
114	PLG	Hydrolase; protease
115	PNPO	Oxidoreductase
116	PON1	None
117	POR	None
118	PPARA	Nucleic acid binding; receptor; transcription factor
119	PPARD	Nucleic acid binding; receptor; transcription factor
120	PPARG	Nucleic acid binding; receptor; transcription factor
121	PPIB	None
122	PRDX2	Oxidoreductase
123	PRKACA	None
124	PROCR	Enzyme modulator; receptor
125	QDPR	Oxidoreductase
126	RBP4	Transfer/carrier protein
127	REN	Hydrolase; protease
128	RXRA	Nucleic acid binding; receptor; transcription factor
129	SEC14L2	None
130	SHBG	None
131	SLC9A3R1	None
132	SOD1	Oxidoreductase
133	SRC	None
134	STS	Hydrolase
135	SULT2A1	None
136	THRA	Nucleic acid binding; receptor; transcription factor
137	THRB	Nucleic acid binding; receptor; transcription factor
138	TNF	Signaling molecule
139	TPI1	Isomerase
140	TRAF2	Signaling molecule
141	TTPA	Transfer/carrier protein
142	TTR	Transfer/carrier protein; transporter
143	TYMP	Transferase
144	VDR	Nucleic acid binding; receptor; transcription factor

## Data Availability

All data are available from the corresponding author upon reasonable request.

## Abbreviations

DR: *Drynariae Rhizoma*
GO: Gene ontology
TCMSP: Traditional Chinese Medicine Systems Pharmacology
OB: Oral bioavailability
DL: Druglikeness
CPI: Chemical-protein interactome
PPI: Protein-protein interaction
dock-IN: Combining docking with intelligence
BP: Biological process
CC: Cellular component
MF: Molecular function
DAVID: Database for Annotation, Visualization, and Integrated Discovery.

## Supplementary Materials

Supplementary Materials Supplementary Table 1: the topology properties of active ingredients of DR.
