# Supplementary material for: Identified the Synergistic Mechanism of Drynariae Rhizoma for Treating Fracture Based on Network Pharmacology
Source: Evid Based Complement Alternat Med. 2019 Oct 20;2019:7342635. doi: 10.1155/2019/7342635 (PMC6855049; doi:10.1155/2019/7342635)
Supplement: Supplementary Materials — Supplementary Table 1: the topology properties of active ingredients of DR. [file 7342635.f1.docx]

**Supplementary Table 1.** The topology properties of active ingredients of DR.

| **No** | **Active ingredients** | **Degree** | **Betweenness centrality** | **Closeness centrality** |
| --- | --- | --- | --- | --- |
| 1 | Cyclolaudenol | 70 | 0.133 | 0.494 |
| 2 | cycloartenone | 70 | 0.133 | 0.494 |
| 3 | Cyclolaudenol acetate | 54 | 0.111 | 0.449 |
| 4 | davallioside A_qt | 50 | 0.113 | 0.440 |
| 5 | 22-Stigmasten-3-one | 50 | 0.082 | 0.44 |
| 6 | beta-sitosterol | 47 | 0.067 | 0.432 |
| 7 | Stigmasterol | 45 | 0.051 | 0.428 |
| 8 | Eriodyctiol (flavanone) | 44 | 0.067 | 0.426 |
| 9 | (2R)-5,7-dihydroxy-2-(4-hydroxyphenyl)chroman-4-one | 44 | 0.063 | 0.426 |
| 10 | eriodictyol | 42 | 0.048 | 0.421 |
| 11 | naringenin | 42 | 0.046 | 0.421 |
| 12 | digallate | 40 | 0.153 | 0.417 |
| 13 | Aureusidin | 40 | 0.059 | 0.417 |
| 14 | luteolin | 39 | 0.038 | 0.415 |
| 15 | kaempferol | 34 | 0.030 | 0.404 |
| 16 | (+)-catechin | 33 | 0.033 | 0.402 |
| 17 | xanthogalenol | 29 | 0.048 | 0.394 |
